# Up to the Challenge: Adapting Pediatric Intensive Care During a Global Pandemic

**DOI:** 10.3389/fped.2022.910018

**Published:** 2022-07-13

**Authors:** Myra Pereira, Olugbenga Akinkugbe, Laura Buckley, Elaine Gilfoyle, Sarah Ibrahim, Melissa McCradden, Sarah Somerton, Karen Dryden-Palmer

**Affiliations:** ^1^Department of Critical Care Medicine, The Hospital for Sick Children (SickKids), Toronto, ON, Canada; ^2^SickKids Research Institute, The Hospital for Sick Children (SickKids), Toronto, ON, Canada; ^3^Lawrence S. Bloomberg Faculty of Nursing, Centre for Advancing Collaborative Healthcare and Education (CACHE), University of Toronto, Toronto, ON, Canada; ^4^Department of Paediatrics, Faculty of Medicine, University of Toronto, Toronto, ON, Canada; ^5^Centre for Advancing Collaborative Healthcare & Education (CACHE), University of Toronto, Toronto, ON, Canada; ^6^Department of Bioethics, The Hospital for Sick Children (SickKids), Toronto, ON, Canada; ^7^Dalla Lana School of Public Health, University of Toronto, Toronto, ON, Canada; ^8^Genetics and Genomic Biology Research Program, Peter Gilgan Centre for Research and Learning, The Hospital for Sick Children (SickKids), Toronto, ON, Canada

**Keywords:** pandemic, adult critical care, adaptation, interprofessional care, COVID-19, pediatric intensive care

## Abstract

**Background:**

The COVID-19 pandemic has strained health systems world wide. In our region, surging numbers of critically ill adult patients demanded urgent system-wide responses. During the peak of the pandemic, our Pediatric Intensive Care Unit (PICU) team redesigned the existing educational resources and processes of care to ensure for adult patients for the first time in the hospital’s history.

**Aim:**

Describe the experiences and impacts of the rapidly initiated Adult COVID-19 Program on health care providers (HCP) and family members. Havelock’s Theory of Change framed the examination of Adult COVID-19 Program participant experiences and surfaced lessons learned.

**Materials and Methods:**

A quality improvement review was employed to collect feedback about the program experience from the health care team and patient’s family members. HCP completed a questionnaire 10 months following the implementation of the program and feedback from family members was provided during the program was obtained. Havelock’s Theory of Change was used to explore trends and frame participants’ experiences.

**Results:**

Pediatric Intensive Care Unit bedside team members and clinical leaders (*n* = 17), adult hospital partners (*n* = 3), and family members (*n* = 8) participated. HCP describe; motivation and readiness; concern for personal safety and uncertainty experienced in the early program phases; the importance of supports and resources; use of relationships and collaboration to facilitate change; the emotional impacts of this unique experience; and opportunities for individual and team growth. An overarching theme of ‘doing our part to help’ emerged. Family members described the positive impacts of family-centered interventions offered, individualized care, and shock at their family member’s illness.

**Conclusion:**

The PICU team rapidly adapted to provide care for adults at the peak of the pandemic. Family members expressed feeling grateful for the care their loved ones received in the pediatric setting. The experience of caring for adult patients with COVID-19 was a source of tension, personal growth, and meaning for the pediatric intensive care team.

## Introduction

The Corona Virus Disease (COVID-19) pandemic has strained health systems due to unprecedented numbers of patients requiring Intensive Care Unit (ICU)-level care and systems were overwhelmed. In March of 2021, Ontario critical care units were at 180% of the pre-pandemic occupancy (1). Strategy involving the redistribution of hospital resources to critical care areas and upskilling non-critical care providers to care for critically ill patients were activated. In contrast, the demand for pediatric critical care services was uncharacteristically low. COVID-19 infection rates were two times lower in children than in adults and children demonstrated a lower likelihood of requiring ICU-level care (2). Suspension of elective admissions coupled with the impact of pandemic “stay at home” precautions also contributed to a lower than typical hospital admission rate for children. Regional healthcare leadership convened a Command Table to direct critical care resources and activities during the crisis and determined that pediatric critical care services would be extended for adult patients with COVID-19. For the first time in the hospital’s history, adult patients were cared for in this stand-alone pediatric tertiary/quaternary center. Adult patients were selected on a case-by-case basis, for limited co-morbidities and age of less than 50 years to reduce the need to consult adult specialists and align as closely as possible with the expertise of the pediatric intensive care unit (PICU) team. Partnering adult specialists from nearby hospitals were available for daily review of patients and consultation as needed. The impact on health care providers (HCP) required to rapidly uptake new knowledge, new tools, and new ways of working in the context of uncertainty and change is unknown. Appreciating that this was a rapidly developed initiative, this paper describes the experiences of HCP and family members participating in the Adult COVID-19 program. Havelock’s Change theory was used to frame the exploration of participant experiences to this rapid change and adaptation to caring for adult patients with COVID-19.

## Materials and Methods

### Design

An exploratory quality improvement review was employed. The project was approved by the Quality and Risk Management Department at the Hospital for Sick Children (SickKids), the largest pediatric health care center in Canada and a leading international pediatric hospital (3).

### Setting

The PICU at Sick Kids Hospital can accommodate up to 20 patients. The PICU alongside the Cardiac Critical Care Unit is one of two units integrated into the Department of Critical Care Medicine. The PICU team had expertise in caring for young adults to the age of 18 years and substantial experience with the life-sustaining technologies required for COVID-19 support [i.e., continuous renal replacement therapies (CRRT), extracorporeal life support (ECLS)], which supported the short 10-day period to prepare for adult patients. Critical care service for children needed to remain undisrupted, therefore limiting adult patients to 8 and dedicating resources to each specific population helped maintain the capacity to provide uninterrupted service to pediatric patients.

### Data Collection

Data were collected using a survey questionnaire comprised of 7 items designed to prompt reflection on the scope of the experience including; preparation for adult patients, providing care to adults, challenges in care delivery, perspectives on the support and resources offered through the program, social and emotional impacts and any learning gained from the experience.

The questionnaire was designed by the study team and was informed by Havelock’s Theory of Change. Havelock identifies that the relationship with the system for change, diagnosis of the need for change, acquisition of resources for change, a purposeful section of a pathway for change, establishing and accepting the change, and maintenance of or separation from the change are the progressive elements of the change process. These elements can occur at the organizational and individual levels (4).

The questionnaire was piloted for face validity and feasibility with a representative member of each of the participant groups (bedside team, clinical leadership, adult HCP partners) and a single minor grammatical revision was made to the items. Recruitment was by word of mouth and enrollment continued until no new items were identified in the responses. The questionnaires took approximately 15 min to complete and were administered based on the participant’s preference, either face to face or *via* telephone. Participant responses were transcribed at the time the questionnaire was delivered and read back to each participant for validation and clarification. Data from HCP were collected 10 months after the Adult COVID-19 program. Family member perspectives were collected from emails and written correspondence sent to the team during the Adult COVID-19 program. Questionnaire items are listed in Box 1.

**Box 1. Stem questions for adult COVID-19 program provider feedback.**

1. What were your first thoughts when hearing about the adult COVID-19 program?

2. What challenges did you experience once the program was launched?

3. What resources were available to address these challenges and were they effective? What communication strategies were effective or ineffective?

4. What team changes/pivots/adaptations were effective and why?

5. What was caring for adults like for you?

6. What was your personal take away(s) from the experience?

7. Is there anything else you would like to talk about that we did not address?

### Participants

Interprofessional HCP who provided clinical care to patients, leadership during the Adult COVID-19 Program, or served as adult HCP consultants during the program were eligible. Purposeful sampling was carried out for a broad representation of the disciplines, roles, and experiences of participants. Family members of adult patients cared for in the PICU during the program who provided written feedback about their experience to the team comprised the family sample.

### Data Analysis

A narrative review was conducted using a constant comparative approach. Two study team members performed the initial analysis. Provider responses, stratified by role (bedside nurses, clinical leadership, IP team members, and program leadership) were compared and contrasted to surface common provider experiences. Family member feedback was unsolicited and offered during the program interval. Emerging items and trends were reviewed by four study team members. The final items were appraised using Havelock’s Theory of Change framework (4).

## Results

The program was active for 10-weeks between April and June 2021. A total of 425 adult patient days were completed. Feedback was obtained from bedside staff (*n* = 8), clinical leaders such as program directors, advanced practice nurses, and educators (*n* = 9), and adult hospital HCP (*n* = 3) for a total sample of 20. Eight patient family members provided feedback. HCP experiences focused on motivation and readiness for adult care, personal safety, uncertainty, support and resources, relationships and collaboration, emotional impact, and opportunities for growth. Items surfaced by family members included family inclusion, individualized patient care, and shock at the severity of their loved ones’ COVID-19 illness.

### Health Care Provider Experiences

#### Motivation and Readiness

Health care providers described both enthusiasm and concern when considering the onset of the program. Some HCP with previous experience caring for adults expressed enthusiasm to use this skill set again, for example, one bedside nurse stated “I thought it was awesome! I had experience caring for adults.” Many members of the registered respiratory therapy (RRT) group had current adult clinical care experience and were positioned to help. An RRT explained, “I wasn’t overly concerned, I had some experience working with adults as an RRT student and in my first year in practice as an RRT.”

There was also confidence in the team’s ability to manage the challenges of adult care. Many HCP looked to other challenges and successes as proof of a collective ability for rapid change. A charge nurse commented, “It needs to be done, we have the capacity to do it, bed space, nurses available, as well as the skill of our team in pulling together quickly and doing what needs to be done, even with very limited experience.”

Some individuals felt concerned about their own skills and the team’s collective ability to care for adults. One bedside nurse shared; “I worried about taking care of adults, we have no experience with medications or ventilation of adults… I wondered if we were doing these patients wrong and if they would be at risk.” Comments were shared about anxiety related to the acuity and the physical demands of larger patients. One bedside nurse summarized this aspect of the experience by saying, “I really worried about the level of illness and acuity and was expecting unpredictability and co-morbidities that were not familiar.” An IP team member added that “The needs of these larger and mature patients were very different and could not be anticipated.” Many expressed anxieties that they would be able to acclimatize, and feared making medication errors or missing essential clinical cues. One bedside nurse described this concern, “I was initially anxious about my skills for providing care to this population.” Another bedside nurse expressed, “I was concerned about the level of illness and acuity, unpredictability, and co-morbidities that are not familiar.”

#### Personal Safety

Bedside HCP initially had concern for their personal safety related to increased exposure to patients with infectious COVID-19. The risk of transmission to staff from patients and uncertainty about best infection control practices were highlighted as particular stressors at the beginning of the program. A number of participants noted that the addition of a dedicated Personal Protective Equipment (PPE) coach role to the clinical area did much to mitigate this concern and supported confidence in the procedures protecting staff. One HCP recounted, “The implementation of the PPE coach really helped decrease anxiety around proper PPE use and maintaining our own safety and wellness.” This was perceived as an organizational investment in worker safety and supportive of quality patient care. A clinical leader shared, “Throughout the pandemic thus far, we were shielded from exposure risks at work and this is what it has been like for other adult ICUs.”

Best practices for infection control were refined and clarified over time and PPE supplies were secured. Participants acknowledged achieving these sureties decreased their concerns. A clinical leader noted, “The fear of COVID exposure decreased over time as no bad HCP outcomes were experienced and we got into the groove of COVID care.”

#### Uncertainty

Health care providers identified that uncertainty about how the program would operationalize the needs of the patients and the resource availability played an initial part in their adaptation to caring for adults. Some HCP noted that over time they settled into predictable routines and expectations when caring for adult patients. The dynamic and iterative progress of the program was acknowledged and a bedside nurse summarized, “The challenges we faced were mostly at the beginning and rapidly improved after a brief learning curve.” Another bedside nurse noted,

“Although I was initially anxious about my skills for providing care to this population, I feel like I was well supported in gaining the additional learning. I really felt like myself and the team found our groove and ended up not only providing excellent care but enjoying the experience.”

Leaders reported that the demands on their roles amplified with the decision to provide care to adults. Priority concerns emerged for them related to maintaining team cohesiveness, being visible to the team, and addressing the uncertainty that was being experienced. Leaders also noted feeling pressure to act quickly and definitively to respond to the urgency for increased adult critical care capacity. A program leader reflected on the challenges of keeping a large number of clinicians and stakeholder groups aligned to the program’s purpose; “Creating a unity of thought and unity of action was difficult. It was challenging but essential to consistently create things.” Another clinical leader initially questioned, “How are we going to pull this together in such a short time?”

Many individuals commented on the positive impact of visible leadership presence on their feelings of uncertainty. Ready access to leaders, in turn, supported interprofessional coordination and problem-solving. Leadership interventions that bedside staff identified as mitigating their uncertainty were daily program summary emails, attendance to adult rounds, daily program huddles, and transparency about the “behind the scenes” preparations provided to the team in real time.

#### Supports and Resources

The short timeline for launching the program raised many concerns that the preparation of the environment, resources, team, and organizational infrastructure would not be adequate. Shift working HCP are not at the hospital every day and the already short preparation time was perceived as much shorter in this group. A bedside nurse who was off shift during the preparations stated, “I felt it all happened very fast- we got a quick in-service and some emails on how to ventilate COVID-19 adults. It turns out that they weren’t just big kids-they’re very different and there was a lot of learning!”

Health care providers also noted that much of what they understood about pediatric critical care was applicable to caring for adults. Some staff articulated a feeling of confidence in their ability to care for adults, “I felt positive about my abilities and the utility of my skills.” Skills identified as transferable and readily adaptable to adult care included; care coordination, ventilation, managing CRRT, and ECMO circuits, and sedation and pain management. HCP acknowledged the preparation and resources available to support the team to adapt were dynamic and evolving across the trajectory of the program. One bedside nurse stating, “I feel like things were changing daily.” An IP team member noted that the preparations were intended to provide a basis from which iterative adaptation would emerge, “The goal was to build a foundation that would be revised and adapted as we gained experience and knowledge, we drew in new resources as needed.”

Some HCP reported confusion and unease with some of the care processes that evolved. A bedside nurse with adult experience shared frustration, “… medical care did not align with my experience, for example, sedation management, it felt that too many drugs were used too quickly.”

Both bedside staff and clinical leadership acknowledged the extensive learning that was required to care for high-acuity adult patients that took place. One bedside nurse noted, “We knew that these patients could rapidly decline, but we were still not mentally prepared for it. We didn’t anticipate the need to prepare in advance for the rapid progression of the disease.”

Adult partners that had faced clinical resource shortages for months, reflected on the prospect of partnering with the PICU and the addition of the organization’s resources for care of the growing number of patients;

“We were happy to partner with SickKids when they sent ICU staff to help with our COVID patients. For our multi-disciplinary team, it was a huge lift in morale when SickKids then partnered with us to take on high acuity ICU patients.”

Tangible resources offered to support the program were noted by bedside staff, IP, and leadership members as particularly impactful for both practical reasons and as representative of the hospital support. A leadership team member stated, “Bringing on additional team members, including new roles [personal support workers (PSW) and clinical externs] to help with the physical needs of these patients. This significantly alleviated the physical burden placed on the nurses that the organization recognized.” A bedside nurse described the positive impact of the new roles introduced and that they were continued after the program,

‘The addition of the PSW was an adaptation that was not only useful in the moment but has now introduced us to a new role that we can continue to implement in PICU. Most of us had never worked with a PSW before.”

A Clinical leader stated that, “…it felt like an organizational wide effort, I had no apprehension about the organization committing to help.”

Some staff did note that the organization’s response to the pandemic was problematic. A clinical leader stated that

“The work from home situation for key stakeholders was negative in the experience. Having the infection control team not on site while we were working out the kinks caused a lot of uncertainty and angst that might have been avoided.”

An IP team member noted that support from the organization to prioritize program preparations, and enabled focus. “Personal needs and goals” were suspended and individuals were absolved of other pressures while developing the program. Redirecting resources and the team’s attention to this program was essential in achieving the objectives necessary to support the team and these patients.

Some participants voiced concern that the level of organizational support would not be sustained after the program’s closure. A clinical leader characterized organizational support for the PICU as a temporary commitment questioning if it would be sustained,

“Why was the hospital responding now to our needs when in the past we had slow or no response in regular times? For example, the increased supply of pillows. We were always asking before for pillows and never got them and now we have so many pillows we were drowning in them.”

A bedside nurse noted that “the changes and resources are now gone, disappeared, and our needs will be unheard again.” Another clinical leader further acknowledged, “The urgency of the situation proved that there is room to improve our current efficiency when introducing change. There are ways to do things faster if you need to get things done.”

#### Relationships and Collaboration

Relationships and resulting collaborations featured in the HCP experience. IP coordination of care was perceived to be essential in meeting day-to-day patient needs. The imperative to preserve pediatric patient service during the program interval resulted in the emergence of a dedicated team for adult care. Adjusting work flows specific to adult patients with the addition of senior leadership and adult specialist participation, collaborating on the timing of individual patient treatments (medications and ECLS circuit interventions), and launching a dedicated process for mobilizing and turning adult patients were noted as strategies that promoted a sense of community around these very demanding tasks. A bedside nurse shared,

“Excellent teamwork when it came to group tasks such as proning. We learned that adult care was more of a ‘team sport’ (often due to size) so we implemented strategies to support this, like scheduling proning times so the team could flip each patient in order.”

An IP team member noted that these coordinated approaches and “continuing the focus were a work in progress throughout.” A number of participants identified the ongoing hurdles and team discussions for coordinating care were valuable points of connection. One IP provider stated, “The twice weekly (adult program) IP rounds for coordinating care and managing with existing demands and caseloads in the hospital was key.”

Existing relationships amongst the team were leveraged for managing adult patient needs. Some bedside nurses felt the adult-focused respiratory training modules did not meet their needs. The RRT educator responded by very quickly by creating a video learning module to address the remaining knowledge gaps. A clinical leader shared about the integration of team members with adult experience describing, “tapping into skill sets of team members who have cross trained in adults and this was hugely helpful. I found the RRTs were an excellent resource in particular, and they stepped up and provided ‘just in time’ training to the team.” IP team members adapted through continued collaboration within their disciplinary communities within and outside the hospital. One IP team member noted, “Ongoing communication within our disciplinary group was essential.”

This reliance on known internal experts was augmented by relationships with experts outside the organization. The direction provided by adult partners from adjacent adult hospitals was seen as essential in providing direction in the day-to-day care and problem-solving for specific clinical challenges. Participants noted that adult HCP provided support for adult-specific expertise attended rounds and patient conferences when required and provided the PICU team with 24-7 on-call accessibility. Similarly, partnerships were developed with adult experts in other disciplinary fields. A clinical leader stated, “Nursing leadership from [adult hospital] and [second adult hospital] proved invaluable. Lots of helpful information was shared from their teams.” A clinical leader shared,

“I found it extremely helpful to connect with our adult care partners…. They brought me to their ICU so I could physically see how they were providing care to these patients, what equipment they were using, and what care strategies they had found most effective. The adult palliative care provider was very useful to assist us with family follow up and linking to adult based resources.”

When participants were asked what personal learning they gained from the experience answers were focused on the performance of the team and the sense of pride at having done very challenging work. “I learned that we have a great team that was able to come together in a crisis and in completely new situations.” A program leader noted “The immense efforts of the entire hospital to make this possible, and the pride in the PICU of their efforts. I hope this message doesn’t leave the PICU.” A sense of awareness of team resilience and ability was echoed from the bedside team and program leadership levels. A program leader reflected on how the experience changed her level of appreciation for the team, “I have a much better appreciation of what we are capable of. I am proud of our entire team and I think it brought all of us closer together.” A bedside nurse commented, “We are more flexible and adaptable than we would have thought possible.”

Bedside staff perception of relationships with patients and families surfaced issues related to pandemic restrictions on the family presence and the innovations developed to bridge those absences. The provision of family-centered care (FCC) aligned with practices established with pediatric patients and their families was not universally embraced by the HCP team. A front-line nurse noted that “100% pediatric principles of [pediatric family-centered] care were transferable to these patients,” while another stated, “I don’t think FCC applied to these patients as family members were not present.”

One adult partner shared the value of transferable FCC principles and discussed the impacts their team observed after transferring back to the adult setting:

‘Families provided feedback about the deep love and care they receive daily from Sick Kids. It brought me to tears, in 4 different conversations today, to hear the complete transformation of their story, independent of the outcomes that they’ve experienced with your team. Thinking about what we are capable of as a team, society, and as humans, tonight reflecting on their words brings hope and light back into a heart that was starting to feel it fade this year.’

Feedback was mixed when describing the use of technology to maintain connections between family members, the patients, and the care team. A clinical leader noted, “Using virtual technology was an excellent adaptation that allowed us to link patients and families, and provide FCC even with the extreme visiting restrictions.” Technical issues with the web-based tools for family engagement were reported as a source of frustration to achieve best practices in FCC. One bedside nurse noted that “The patients were too busy and we didn’t have time to trouble-shoot the iPad for a family visit, it just didn’t work.” Another bedside nurse noted, “The extended team was helpful to provide this support to families; the bedside nurses just couldn’t do it and it was reassuring to know that it was being attended to.”

Perceived benefits of limited family presence were noted. A clinical leader recalled the limited family visitation helped preserve clinician attention to the high-acuity patient’s needs, “The limited family presence was protective and eased stress as no family interfacing.” An IP team member noted how their work processes had to be adapted to meet the needs of the new adult patient population, “There was an adjustment period to learn how to respond to the complexities of the adult’s family situations. I was not used to working with spouses, adult children, and substitute decision makers who were not related to the patient.”

#### Emotional Impacts

The moral and emotional impacts on staff surfaced in the feedback and included a renewed sense of purpose, pride in the team, and the consolidation of meaning for HCP. One bedside nurse described a sense of relief to finally be active in the pandemic efforts, “When I first heard about this I thought ‘We should do this- we should help,’ we need to look at the broader picture outside of Sick Kids.” A member of the clinical leadership team also noted, “I was excited to be able to help with the COVID-19 response effort. Prior to this, we didn’t feel as though we were contributing fully because we had not had many pediatric cases in PICU.”

Health care providers reported a sense of accomplishment for their contributions to the program and to pandemic efforts at large, in particular the value of their skill and knowledge. One bedside nurse commented that she learned to, “feel positive about my abilities and the utility of my skills.” Another commented that,

“I enjoyed the challenge and stretch to my learning. It was rewarding because you know it was good work. Many reported they felt that patients received excellent care and that they knew family was grateful, the patients were well cared for, it was rewarding.”

For some, the experience consolidated their choice of medical specialty. A bedside nurse noted that this experience, “…helped me to realize I am in the right place and pediatrics is what I want.” Others recalled that the work was hard and, ‘…the novelty wore off…and we acclimatized to the pace and demands pretty quickly.” A Clinical Nurse Specialist described feeling renewed passion for clinical care,

“In my role, I don’t often get to have as much hands-on time with the patients, but because of the physical demands of the patients, and the novelty of some of the procedures (e.g., “jelly roll” proning), I was able to spend much more time in the patient rooms than usual.”

There was recognition by HCP that the emotional content of working with adults was different, especially as it related to patient advocacy and end of life situations. Navigating the substitute decision-maker process was identified as an unexpected challenge. Similarly, navigating end-of-life for adult responsibilities (family income earners and child rearing) was perceived as different them the experience of childhood death. One bedside nurse summed this up by stating, “Caring for adults was different than I expected. Seeing people my own age become critically ill and die was very scary and sad in a different way than I was used to.”

Some mentioned tensions related to the nature of the program as a “stop along the way” and the impact of not knowing what happened to these patients they had invested so much in. One IP provider stated, “There was a different tone of care as we were a stop-off and not really a part of the continuity of the recovery, that was sad for me.” A clinical leader also noted that “It would be good to know patient outcomes or hear about family satisfaction. The lack of closure is hard, I sometimes wonder if families get follow up for bereavement.”

When considering the specific emotional and mental health resources that were initiated as part of the program one Clinical Leader reflected on their experience of “…reassuring people that the care they were providing was equal elsewhere. We need a better way of supporting people when they (feel) they are not meeting their own expectations.” One adult HCP partner shared feedback from patients after returning to adult centers,

“For the few patients who were transferred back from SickKids, the feedback from families was that SickKids team was compassionate, diligent in their approach, and were doing their best to keep families informed given visiting restrictions.”

#### Opportunities for Growth

Personal and team growth from the experience was acknowledged. As this clinical leader stated, “We adapt very well- our culture was already situated to make us successful.” A number of team members reported that the opportunity to expand boundaries and develop new innovations was invigorating and that seeing beyond our previous rules/limitations and boundaries was a positive aspect of the experience. This participant sums up the experience of growth:

‘The process of bringing [in] adult patients, especially in the preparation phase, was incredibly exhausting, but ultimately, it was something that we recognized needed to be done and it was great to see the team pull together. It was also gratifying to have the rest of the hospital, and even the larger community recognize the work that we had done to make this all happen.’

### Family Experiences

#### Family Inclusion

In their feedback to the PICU team, family members reflected on the care their family members received as being compassionate and family-centered. Families did not provide any comments on the fact that their adult relative was receiving care in a pediatric hospital. Family members valued the extension and adaptation of pediatric family-centered care interventions and describe these efforts as a source of connection, coping, and meaning during the patient’s illness. Comments focused on family perceptions of empathy and the positive value of the family-centered care interventions they received, such as video calls, daily updates from the medical team, and efforts to personalize the patient environment with music and patient diaries.

One sibling of a patient wrote, “At a time where our family became helpless, the support that your team provided us was beyond anything we could have imagined. We will forever be grateful for the care, kindness, and humanity that was provided.”

Some family members expressed the significant impact of the family-centered interventions in particular the adaptations to facilitate family engagement and family presence when possible. The adult child of a patient who died shared,

“Thank you so so so much for putting in the exception for me to come and visit as well as my sister, being able to come and see my Dad and spend time with him has made these last couple of weeks so much easier.”

#### Individualized Patient Care

Families expressed that the fundamental family-centered care principles aligned with their individual, cultural and faith needs, for example, providing family-specific spiritual resources. Further interventions of frequent contacts to home with video calls, unit video tours for orientation, and facilitating community connections where possible were noted as impactful. A family member shared,

“My parents continue to be in touch with the [spiritual agency], who are including [sibling] in their prayers daily. Thank you so much for arranging this for us, we had no idea programs like this were even available. This has been very helpful for my parents to cope, and continue to heal through their faith.”

A family member of a patient who passed away after repatriation to an adult hospital shared,

“In behalf of [patient], I would like thank each and every one of you at SickKids who took really good care of [patient]. I really appreciate how you all cared for him for a month or so and for all the support you all have given to me and [patient’s children].”

#### Shock at the Illness

Many family members wrote about their shock at a family member becoming so ill and expressed a need for the staff to know the patient had taken precautions to avoid the virus, fearing their loved one might be blamed for their illness. A sibling of a patient wrote,

“COVID-19 hit our family very hard, with both my [siblings] and I ending up in the hospital. We would never have imagined this would have happened to us, we were so careful, we followed every protocol, took every precaution. Just really goes to show this virus does not show any mercy, everybody needs to be mindful and cautious.”

## Discussion

Admitting adult patients with COVID-19 to the PICU at SickKids proved to be a demanding learning experience that balanced both concern for and excitement about contributing to the pandemic response. A core principle of pandemic responsiveness is solidarity and senior clinical leadership felt an imperative to assist adult critical care colleagues and share the burden of the influx of critically ill adult patients (5). Informal consultations with the Bioethics team supported this decision. This solidarity felt by HCP extended beyond hospital boundaries, disciplinary domains, and geography such that it was an essential motivator for initiating the Adult COVID-19 Program.

According to Havelock’s Theory of Change, change is conceptualized as relational and requires facilitation in the form of frequent communication, updates, rewards, personal recognition, and adaptation (4). Within the initial stages of change, Havelock highlights the importance of building relationships within a system that can identify and evaluate the need for a change (4). During the adult COVID-19 program, relationship-building strategies were advantageous to quickly identify and address concerns in the development and early implementation phases of this program (4). Interventions aimed at increasing senior leadership visibility, leveraging existing learning resources, and connecting the team with adult content experts, were perceived by HCP as effective in reducing uncertainty and building interpersonal and team linkages. PICUs in England that took on adult patients during the COVID-19 pandemic also describe their successes in using leadership presence and optimizing effective communication to nurture relationships and support frequent program changes (6). The contrast in provider reports in this initial stage (readiness vs. unprepared), may be indicative of varying exposure to relationship-building efforts or reflect how prior experience with this novel patient population played a role in motivating HCP to accept the change. Individual perceptions of competency were threatened for some by the pace and iterative nature of program development thus heightening their uncertainty. The significant structural and process changes required created tension and concern for the competence of pediatric experts caring for an adult population. The context of the team’s limited experience with COVID-19 and the rapidly changing landscape of best practices in COVID-19 treatment added additional pressures in this regard.

Other relational tensions were related to family-centered care in the context of adult patients and COVID-19 visitor restrictions. FCC approaches were characterized by HCP as greater than simply facilitating visiting and information sharing about the patient. Achieving FCC for adult patients required innovation and dedicated resources to facilitate interventions such as virtual visits, family orientation and to mark the story of the patient’s illness journey. Virtual visits were seen as beneficial for communication and orientation activities and also provided an opportunity for family members to make sense of their own emotions and coping (7). Typically, facilitating FCC would fall to bedside nursing staff. In this situation, the high illness acuity and patient workload consumed the majority of that nursing care time. While necessary because of pandemic recommendations, limiting visitors for patients in the hospital raised concerns within the team that this might negatively impact patient outcomes (8). Family members’ appreciation of the care their loved ones received in this alternate setting is in contrast to these HCP concerns.

Havelock’s Diagnosis element of early change was reflected in the continuous evaluation of new processes, tools, resources, and team impacts (4). These were seen by participants as effective strategies in the context of this rapid change (9). Leadership approaches that engaged and readily addressed bedside staff concerns facilitated rapid adaption and iterative learning (10). This continuous attention to evaluation also served to maintain situational awareness. Access to information through established and trusted channels (email, huddles, and rounds) was facilitative of change according to HCP. Open communication about the full scope of the program and the regional pandemic situation provided bedside staff with reassurance that all elements of care delivery were being attended to and connected staff to these efforts when individuals were not onsite.

Havelock’s third element for change is acquiring resources for the change (4). Acquiring resources and developing solutions forms new practices, solidifies new relationships, addresses education gaps, and sources resources like equipment and supplies. These activities played an essential role in supporting successful patient care according to HCP. For example, perceived risks to personal safety were addressed by maintaining resources for protective strategies and adding new resources (PPE coach) resulting in HCP acclimatization to the new protocols. Expansion of the HCP roster to include clinical externs and PSWs helped to distribute patient care responsibilities and was a well-received change among the interprofessional staff. Optimizing interprofessional roles helped to support FCC, facilitating connections for patients and their loved ones. The positive feedback received during the program from families about the benefit of FCC interventions reinforced the importance and the value of these activities so that resources were prioritized to ensure this continued.

Stage 4 of Havelock’s Theory of Change focuses on selecting a pathway to operationalize change (4). Rapid knowledge dissemination and information sharing between and within the team allowed for iterative and collaborative problem-solving. Bedside staff shared that the resources and tools were better utilized by participants when resources were matched to their needs. Bi-directional and ongoing evaluation and communication of the needs of the provider team is recommended to sustain high-quality care delivery in the context of rapid change (11, 12).

Accessibility of practical information for bedside staff and ongoing connections with knowledge experts at collaborating hospitals facilitated rapid knowledge transfer and uptake. A collaborative relationship between pediatric and adult care centers was seen as beneficial during rapid program set up, for active care delivery, and for coordinating the transfer of patients back to the adult system. Other centers that adapted PICUs to care for adult COVID-19 patients also reported the benefit of well-established communication and work flows between adult-care and pediatric experts (13).

Interprofessional collaboration mechanisms were seen as an adaptive resource for meeting the needs of HCP and patients within the evolving program. Daily collaborative problem-solving interactions integrated leaders in a continuous process of relationship-building across all program stakeholders. This continuous engagement, shared reflection, and adaptation became part of the adult program team culture. Front-line staff was key in identifying and raising problems, and effective leadership was essential to resolve them. Continuous improvement processes enhanced personal motivations and led to an acceptance of the need for dynamic, ongoing change.

The contrasting staff perspectives related to identifying and acquiring resources could result from the large variation in personal experience caring for adults within each disciplinary group and individual learning receptivity and styles. Interestingly, this rapid change was contrasted with the usual time-consuming nature of organizational change processes when participants described organizational support for the program. The observation comparing how needs were addressed prior to the adult program and during the program may reflect a preference for continuing the rapid change process for future patient care challenges.

In the 5th stage of Havelock’s Theory of Change; establish and accept, clinicians and leaders revealed feelings of solidarity, high levels of interprofessional collaboration, an atypical separation between the team and the patient’s family members, and the emotional burden for clinicians. The experience of solidarity was contrasted with the early feelings of helplessness during the first and second COVID waves as clinicians witnessed their adult colleagues manage the influx of patients. While this strong sense of solidarity was highly motivating, in actualizing these efforts, HCP did face several ethical and emotional tensions at the closure of the program. Bedside staff delivering end-of-life care for adult patients reported a different level of psychological burden in caring for patients who were similar in age. Wellness resources for HCP were implemented during the program, however, the unique experience of identifying closely with patients and families and the known high mortality rates may have increased distress levels when working with this patient group.

The desire to provide “good care” superseded fears for self and extended to a desire to help and to be the right group of people to provide that help. A sense of disconnection from outcomes of survivors created a desire for more complete closure in clinicians. As patients were discharged to other centers for continued recovery the team had a limited ability to self-appraise their efforts and investment in the patients and the program. Anticipating and preparing supportive structures that facilitate coping in the face of fear for personal safety, high patient acuity, and episodic high intensity/short duration relationships with patients may mitigate this in the future.

Havelock’s Theory identifies the 6th and last element of change as maintenance and separation. This involves the integration of change into normal practice and the sustainment of those changes (4). While the adult program was closed as the regional demand for critical care diminished, the team acknowledged maintaining some transferable knowledge and intent to carry this new knowledge onward in pediatric practice. These include clinical gains like procedures to prone larger patients, intramural transport of larger patients, delirium management for older patients, rapid solution-building team processes, and remote family presence and engagement strategies. The versatility and transferability of adult care experiences are perceived as extendable to the care of young adult patients in the future.

This novel program situated the PICU as a part of the greater COVID-19 response network. Adult hospital program leaders echoed the value of the relationship between hospital teams. Clinical Nurse Specialists, Pharmacists, Social Workers, and other allied staff have established stronger relationships with adult hospital colleagues. The experience was characterized by HCP as stressful, uncertain, capacity building for individuals and teams, community building, and consolidating of professional choices and identities. HCP were conscious of their collective contribution to the pandemic response and proud of the rapid learning and adaption they achieved.

## Limitations

This retrospective exploration of a high-stakes and rapidly evolving program pivot in a single center context may not be transferable to other rapid change programs. The program impacts identified are specific to this pediatric hospital system.

The complexity of the program and the short time frame for the program limited opportunities to explore and evaluate specific change interventions within the program and thus may limit the generalizability of the findings. Purposeful sampling for individual participants representative of the IP team may not be an exhaustive representation of the breadth of provider experiences. The inclusion of family feedback provides context for the program and the HCP perspectives. This feedback was not solicited and therefore may not be representative of the experience of all familie.

## Conclusion

This quality improvement study explores the potential impacts of rapid change on health care teams and the factors that facilitate or hinder successful change management. Havelock’s Theory of Change framed the examination of how the PICU team adapted to deliver high-acuity family-centered care for a novel population. Utilizing strategies focused on communication and leveraging interprofessional team expertise maximized system flexibility and enhanced the team’s ability to pivot to the needs of a new patient group. The experience of COVID-19 adult care has inspired questions about how change processes might be improved and surfaced expected and unexpected impacts of rapid change on HCP.

## Data Availability Statement

The raw data supporting the conclusions of this article will be made available by the authors, without undue reservation.

## Ethics Statement

Ethical review and approval was not required for the study on human participants in accordance with the local legislation and institutional requirements. Written informed consent from the patients/participants or patients/participants legal guardian/next of kin was not required to participate in this study in accordance with the national legislation and the institutional requirements. Quality improvement project approval was obtained from the organization.

## Author Contributions

MP and KD-P were the major contributors in developing this manuscript. MP, OA, LB, EG, SI, MM, SS, and KD-P contributed to substantive revisions. All authors contributed to the article and approved the submitted version.

## Conflict of Interest

The authors declare that the research was conducted in the absence of any commercial or financial relationships that could be construed as a potential conflict of interest.

## Publisher’s Note

All claims expressed in this article are solely those of the authors and do not necessarily represent those of their affiliated organizations, or those of the publisher, the editors and the reviewers. Any product that may be evaluated in this article, or claim that may be made by its manufacturer, is not guaranteed or endorsed by the publisher.
